# The Accuracy of a Simple, Low-Cost GPS Data Logger/Receiver to Study Outdoor Human Walking in View of Health and Clinical Studies

**DOI:** 10.1371/journal.pone.0023027

**Published:** 2011-09-13

**Authors:** Bénédicte Noury-Desvaux, Pierre Abraham, Guillaume Mahé, Thomas Sauvaget, Georges Leftheriotis, Alexis Le Faucheur

**Affiliations:** 1 APCoSS, Institute of Physical Education and Sports Sciences (IFEPSA), UCO, Les Ponts de Cé, France; 2 CNRS, UMR6214, Inserm, U771, Medical School, University of Angers, Angers, France; 3 Laboratory of Vascular Investigations and Sports Medicine, University Hospital, Angers, France; Pennington Biomedical Research Center, United States of America

## Abstract

**Introduction:**

Accurate and objective measurements of physical activity and lower-extremity function are important in health and disease monitoring, particularly given the current epidemic of chronic diseases and their related functional impairment.

**Purpose:**

The aim of the present study was to determine the accuracy of a handy (lightweight, small, only one stop/start button) and low-cost (∼$75 with its external antenna) Global Positioning System (GPS) data logger/receiver (the DG100) as a tool to study outdoor human walking in perspective of health and clinical research studies. **Methods.** Healthy subjects performed two experiments that consisted of different prescribed outdoor walking protocols. Experiment 1. We studied the accuracy of the DG100 for detecting bouts of walking and resting. Experiment 2. We studied the accuracy of the DG100 for estimating distances and speeds of walking.

**Results:**

Experiment 1. The performance in the detection of bouts, expressed as the percentage of walking and resting bouts that were correctly detected, was 92.4% [95% Confidence Interval: 90.6–94.3]. Experiment 2. The coefficients of variation [95% Confidence Interval] for the accuracy of estimating the distances and speeds of walking were low: 3.1% [2.9–3.3] and 2.8% [2.6–3.1], respectively.

**Conclusion:**

The DG100 produces acceptable accuracy both in detecting bouts of walking and resting and in estimating distances and speeds of walking during the detected walking bouts. However, before we can confirm that the DG100 can be used to study walking with respect to health and clinical studies, the inter- and intra-DG100 variability should be studied.

**Trial Registration:**

ClinicalTrials.gov NCT00485147

## Introduction

There is much scientific evidence that physical activity or physical fitness can delay or prevent many chronic health conditions [1], and a number of studies show that physical activity or physical fitness are inversely related to morbidity or mortality [1], [2], [3], [4], [5]. As a result of these relationships, as well as the current epidemic of chronic diseases and the high prevalence of physical inactivity, many research studies have been conducted to measure physical activity and to better understand its relationship with health and disease. In addition to measuring physical activity, the evaluation of lower-extremity function is an important goal in the elderly [6] as well as in the management of patients with chronic diseases for both diagnosis and rehabilitation purposes, particularly in relation to cardiovascular heart diseases [7], peripheral artery disease [8] or chronic obstructive pulmonary disease [9]. Lower-extremity function is primarily determined by measuring walking impairment. Thus, for those working in public health, clinical and research settings, accurately measuring physical activity and/or functional limitation is important.

The global positioning system (GPS) is an emerging technique for the study of physical activity [10] and functional limitations [11], [12], [13], [14]. The global positioning system has been recently used to assess walking capacity in patients with multiple sclerosis [13], peripheral artery disease [11], [12] and in patients undergoing spine surgery [14]. However, despite the multitude of potential applications related to the technique, the number of studies using the GPS for health and disease remains very low as compared to the numerous studies that have used other objective measurements of physical activity (*i.e*. accelerometers, pedometers). There are many reasons for this discrepancy, and they are potentially associated: i) the lack of specifically designed validation studies, ii) the dramatic increase of commercially available GPS devices with different costs and unknown accuracy, and iii) the complex operation of most of the currently validated GPS devices, which limit their use by the inexperienced user (such as a patient and/or an older subject) in perspective of large cohort studies.

In our laboratory, we previously showed that the handheld Garmin™ GPS 60 EGNOS-enabled (Garmin Ltd., USA, ∼$200) was accurate in studying outdoor walking in healthy subjects, using a simple data processing [15]. This GPS device has since been used in patients with peripheral artery disease to study walking capacity [11], [12]. Nevertheless, the use of such a GPS device is confined to the research laboratory because the user needs to be somewhat experienced in handling such devices. For example, when using the Garmin™ GPS 60, the user needs to find the correct page displays for the GPS initialization. Moreover, because the configuration (WAAS/EGNOS-enabled option, sampling rate, or data saving/erasing) of the GPS device is performed “on-line” using the appropriate page display, the configuration can be unintentionally modified by the inexperienced user.

Within the last three years, an increasing number of handy and very low-cost GPS data logger/receivers (below $100) have been made commercially available, making them good candidates for large cohort studies. These GPS data logger/receivers are well-suited to this purpose because they are lightweight (below 100 grams), because there is only one start/stop button and because the signal acquisition is given simply by a flashing LED. In addition, all settings and configuration are performed on a personal computer before the measurement. It is currently unknown whether such extremely low-cost GPS data logger/receivers are accurate enough for health and clinical use. To the authors' knowledge, only Townshend *et al.* [16] have published a validation study to measure speed, displacement, and position during human locomotion using a handy (easy to use) low-cost GPS receiver (GPS-BT55, Wonde Proud Technology Co., Ltd., cost approximately $80). However, the GPS that was used was not a data logger, and the data had to be streamed to a phone, thereby limiting the usefulness of the technique. Additionally, the study of Townshend *et al.* was not specifically designed to address the needs of health and/or clinical studies. Indeed, it is an important methodological issue to validate a measurement technique with respect to the final application for which it is proposed. In view of the potential use of the GPS technique for health and clinical studies, there are four important validation steps: i) accurately detecting the physical activity that is being studied (*e.g.*, walking or running), ii) estimating and quantifying the data accurately, iii) reliably showing the relevant parameters associated with the detected physical activity (*e.g.*, walking speed and/or distance), and iv) stating the inter-unit variability of the GPS models that were used.

Our overall aim in this study was to determine the accuracy and the reliability of a simple GPS data logger/receiver (the GlobalSat® DG100) during outdoor walking protocols that were performed by healthy subjects. Specifically, the purpose was to determine whether the GlobalSat® DG100 could accurately detect periods of walking and resting and if it could estimate the speed and distance of the detected walking periods. We hypothesized that the DG100: i) would accurately detects bouts of walking and resting during a series of walking and resting bouts and ii) would accurately estimates the walking distances and speeds of the detected walking periods. To strengthen the study of the accuracy of the GlobalSat® DG100, we simultaneously used the previously validated Garmin™ GPS 60 for direct comparison. This comparison was of particular interest because the required processing methodology of GPS speed signals was validated using the Garmin™ GPS 60 [15], and the external validity (use of the same method with a different device) of this methodology is unknown.

In the present study, we focused only on walking because walking is the most typical human physical activity and has been reported to be a key activity to achieve health benefits [2], [3], [4], [5]. Furthermore, in a clinical context, functional limitations are mainly evaluated through the measurement of walking limitation.

## Methods

### Ethics Statement

This study was approved by our institutional ethics committee (“Comité de Protection des Personnes: OUEST II”) and registered in the American National Institute of Health database under reference n° NCT00485147. For each experiment, the subjects were informed of the experimental procedure, and written informed consent was obtained from all participants.

### Instrumentation

During all experiments, we used the GlobalSat® DG100 GPS data logger/receiver (GlobalSat Technology Corp., Taiwan, cost approximately $60) with its external antenna (AT-65 GPS Active Antenna; GlobalSat Technology Corp., Taiwan, cost approximately $15), and the Garmin™ GPS 60 (Garmin Ltd., USA, cost approximately $200), also with its external antenna (GA 25MCX, Garmin Ltd., USA, cost approximately $30). Throughout the paper, the following abbreviations are used to facilitate reading: “DG100” (GlobalSat® DG-100 GPS) and “GPS60” (Garmin™ GPS 60).

The DG100 is a convenient (see introduction) and inexpensive data logger that includes the “European Geostationary Navigation Overlay Service” (EGNOS) function. Technical details of the DG100 can be found on the manufacturer's website (www.globalsat.com.tw). The GPS60 also has the EGNOS function and has been previously validated to study outdoor human walking [15], and has since been used in clinical studies [11], [12]. However, because of its lack of practicality (see introduction), it cannot be used for large cohort studies. The technical details of the GPS60 can also be found on the manufacturer's website (www.garmin.com). The principles of GPS and EGNOS-enabled GPS have been well-described elsewhere, and to avoid redundancy, we refer readers to the previous articles for detailed explanations [10], [17], [18].

The chosen recording rate for both the DG100 and the GPS60 device was 0.5 Hz, as in our previous studies [11], [12], [15]. During all experiments, both the DG100 and the GPS60 were placed in a backpack, and their antennas were placed over the backpack that was worn by the subjects.

### Experiment 1: accuracy of detecting bouts of walking & resting

#### Objective

In Experiment 1, we explored the accuracy of the DG100 in the detection of bouts of outdoor walking and resting of different durations from prescribed walking protocols (PWPs) performed by healthy subjects. The accuracy of the DG100 was compared to the accuracy of the GPS60.

#### Prescribed walking protocols

Experiment 1 consisted of 10 different PWPs performed at a “usual” walking pace and at a “slow” walking pace, thus resulting in the recording of 20 trials. By performing the 10 PWPs at two different walking paces, we expected to cover a larger range of walking speeds than if we had asked the subjects to walk at only a freely chosen speed. The “usual” pace meant that the subjects were asked to walk at their typical self-selected pace. For the “slow” walking pace, the subjects were asked to walk slower than their usual pace. For both walking paces, the subjects were asked to keep their pace as constant as possible throughout the PWPs.

Each of the 10 PWPs included a randomized succession of 21 bouts of walking alternating with 20 bouts of resting. Based on the results of our previous study [15], our aim was to include a relatively large number of bouts of short duration. Indeed, we showed that these short bouts are responsible for most of the events that were incorrectly detected. Thus, the chosen bout durations of both walking and resting PWPs were 6, 8, 10, 12, 14, 16, 30, 60, 120, 240, 480 & 960 s. Once a random sample of 21 walking and 20 resting durations (different for each PWP) was available, a drawing of lots was performed to define the order of the walking and resting durations for each PWP. Once the PWPs were developed, one of the investigators recorded audio files using an MP3 player. A single audio file recorded the oral directions for the entire duration of each PWP (*e.g.* “you will start soon”, “walk”, “keep walking”, “you will stop soon”, “stop”, and “stay still”) at intervals consistent with each pre-defined PWP.

#### Experimental procedure

Fifteen healthy subjects (F/M = 6/9; 24±7 years, 171±7 cm and 65±5 kg) participated in Experiment 1. Prescribed walking protocols were performed in a designated public park in Angers, France (latitude: 47° 28′ 22″ North; longitude: 0° 32′ 53″ West). This public park is a flat area (no hills) and is free of motorized vehicles, buildings and dense trees. All of the experiments were performed under the supervision of an investigator. Throughout all of the experiments, the investigator waited for the time needed for the two GPS devices to initialize before the experiments could start. The subjects were equipped with the two GPS devices and the MP3 player. The subjects were blind to the PWPs time-course. Throughout the PWPs, the investigator walked ∼10 m behind the subjects and reported any potential external events that could interfere with the PWPs. At the end of the PWPs, the subjects waited for about 2 min (stationary position) and the investigator stopped the GPSs recordings.

#### GPS data processing and analysis

After each experiment, the data were downloaded from the DG100 using the GlobalSat software utility (Data logger PC utility, version 1.1, 2006, Taiwan). The data for the GPS60 were downloaded using the MapSource® software (Version 6, Garmin Ltd., USA). The data were automatically expressed in speed by these programs. The speed values that were obtained were analyzed on a personal computer using a spreadsheet (Microsoft® Excel 2000, Microsoft Corporation, USA) and the specific processing methodology that we had previously validated [15]. Using this methodology with the GPS60, we had previously reported the accuracy of detecting bouts of walking and resting to be 97.1% when only a few bouts of less than 30 s were prescribed in the PWPs. Additionally, the estimation of the distance and speed walked was highly accurate (r = 1 between the actual and the processed distances and r = 0.97 between the actual and the processed speeds).

#### Statistical analyses

To determine the accuracy of the DG100 in detecting bouts of walking and resting and compare it with the accuracy of the GPS60, we performed a “bout-level analysis” that simply relied on the comparison of the prescribed and the detected bouts [15]. Using this procedure, the final analysis was to calculate the accuracy of both the DG100 and the GPS60 in the detection of bouts of walking and resting that were actually performed. For this purpose, the number of incorrectly detected bouts was calculated. A bout was reported to be incorrectly detected if i) one false bout of walking (or more) occurred during an actual bout of resting, ii) one false bout of resting (or more) occurred during an actual bout of walking, iii) the time difference, expressed as a percent error between the actual and the detected bout time, exceeded +/−20%. For instance, if a detected bout that was estimated by analysis to last 8 s actually lasted 14 s, it was reported to be incorrectly detected (error percentage  = −43%). Three investigators performed the GPS analyses and were blind to the time-course of the PWPs and the walking pace modality.

The accuracy of bout detection for each GPS device is reported with 95% Confidence Intervals (95% CI). Between-GPS device comparison of the accuracy of bout detection was performed using the MacNemar test. The percent error for the time difference between the actual and detected bouts (expressed as an absolute value) was also compared between the DG100 and the GPS60. Because the data did not assume a Gaussian distribution, we used the Mann-Whitney test. The statistical analysis was performed with SPSS statistical software (version 17.0, 2008, SPSS Inc., USA). A p-value <0.05 was considered as statistically significant.

### Experiment 2: accuracy of estimating distances and speeds of walking

#### Objective

In Experiment 2, we determined the accuracy of the DG100 in the estimation of outdoor walking distances and speeds from other PWPs performed by healthy subjects.

#### Prescribed walking protocols

Experiment 2 consisted of the recording of an additional 10 PWPs. Each PWP consisted of a series of walking bouts of 50, 100, 150, 200, 250, 300, 350 and 400 m (total distance: 1800 m). The order of the bouts of walking within the PWPs was predetermined randomly. Throughout all the PWPs, each walking bout was separated by a resting period of ∼30 s. The subjects were asked to alternately perform the PWP two times at a “usual” pace and two times at a “slow” pace.

#### Experimental procedure

Ten healthy subjects (32±5 years, 173±8 cm and 67±9 kg) took part in Experiment 2. The prescribed walking protocols were performed on a 400 m outdoor athletic track in Angers, France (latitude: 47° 28′ 22″ North; longitude: 0° 32′ 53″ West). Eight blocks were placed every 50 m on the 400 m outdoor athletic track. The subjects were asked to walk on the interior lane of the athletic track and to stop at the next block when they heard a whistle blown by the investigator. The investigator whistled when the subject was approximately ten meters away from the block where the subject was expected to stop. The actual speed over each bout of walking was calculated by dividing the actual distance traveled by the time measured by chronometry (Geonaute Trt'L 500, Decathlon Ltd., France). For each PWP, the recording was started on the athletic track with the recording of the time and validation of an event marker.

#### GPS data processing and analysis

The GPS data processing and analysis follows the same procedures as described above for Experiment 1.

#### Statistical analysis

The accuracy of the DG100 and the GPS60 measurements was determined by calculating the following for each device according to the statistical procedure proposed by Hopkins [19]: i) the typical error of the estimated accuracy (TEE) and ii) the coefficient of variation of the accuracy (CV). This statistical procedure relied on the comparison of the GPS distances and speeds with the actual distances and speeds. To standardize and facilitate the method of TEE and CV calculations, we used the spreadsheet proposed by Hopkins [20]. Because we also calculated the TEE and CV for each distance (50, 100, etc…, up to 400 m), we could not use the “validity spreadsheet” proposed by Hopkins [21]. As suggested by Hopkins (personal communication), we used “the reliability spreadsheet” and we carefully deleted the term “√2” from the TEE and CV calculation formulas [20]. TEE and CV are presented with 95% Confidence Intervals. Further, the comparison of the CV values was performed for the calculation of the variance of each CV and for the ratio of the larger variance to the smaller variance between the two GPS devices. Finally, we calculated a p-value from the variance ratio according to the F distribution [22]. A p-value <0.05 was considered as statistically significant.

## Results

No external event interfered with any of the experiments performed.

### Experiment 1

We investigated the accuracy of the DG100 for detecting bouts of outdoor walking and resting of different durations from PWPs. The accuracy of the DG100 was compared to the accuracy of the GPS60.

The total recording time that was analyzed for each GPS device was 1540 min (∼25.7 hours). A sufficient number of satellite signals were always available for the DG100 because no GPS signal lost was present in the recordings. Among the 1540 min of recordings for the GPS60, only one episode of undetectable satellite signal was noted lasting 0.97 min. Mean ± standard deviation of walking speed for the DG100 was 2.7±0.4 km/h (range 1.9 to 3.3 km/h) for “slow” pace PWPs, and was 4.6±0.6 km/h (range 3.4 to 5.1 km/h) for “usual” pace PWPs. The accuracy in the detection of bouts for the DG100 was high, 92.4% [90.6%–94.3%]. The accuracy in the detection of bouts for the GPS60 was 77.0% [74.1%–79.8%] and was significantly lower than the accuracy that was reported for the DG100 (p<0.001).

**Figure 1:**
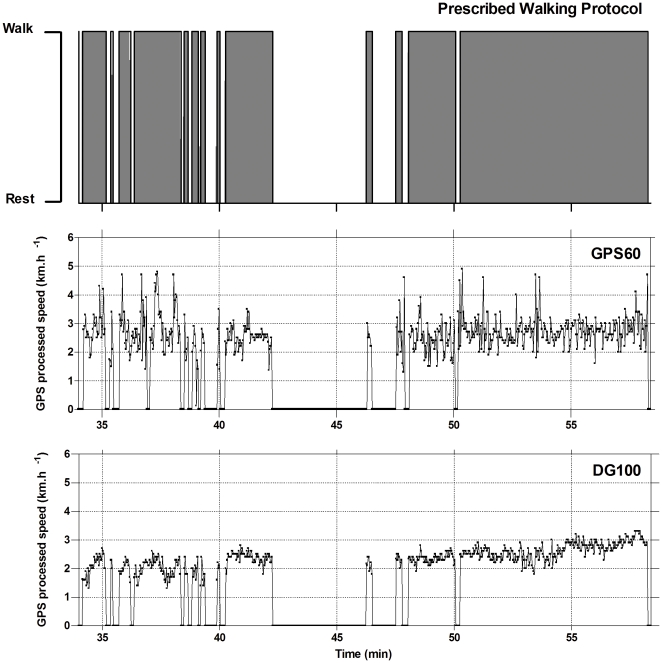
A typical example of GPS processed speed data obtained from a prescribed outdoor walking protocol, both for the DG100 and the GPS60. The entire PWP is not represented on the graph to simplify the figure. The period represented on the graph lasts ∼24 min (from minute 34.2 to minute 58.3).

**Figure 2:**
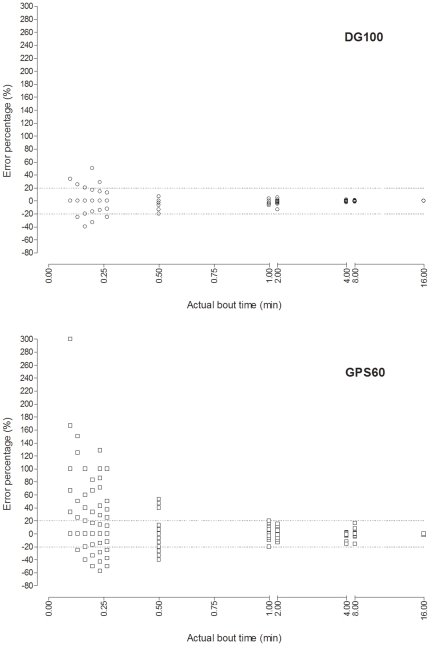
Graphical representation for both the DG100 and the GPS60, of the error percentage for time difference between actual and detected bouts according to actual bout time. Note: concentrations of point near 0% for the DG100 give the impression that there were fewer points, particularly for bouts less than 0.5 min. This was not the case. For instance, there were 65 and 70 bouts of 0.17 min (10 s) for the GPS60 and the DG100, respectivel.

Figure 1 shows a typical example of the GPS-processed speed data that was obtained from the same PWP for each GPS device. It was interesting to observe that the walking speed during the bouts of walking seemed more variable for the GPS60 as compared to the DG100. The graphs in Figure 2 show the percent error for the time difference between the actual and the detected bouts according to the actual bout time for each GPS device. As shown, the percent error increased when the actual bout time decreased. Interestingly, most of the error corresponded to bouts of walking that lasted ≤30 s and especially to bouts that were ≤15 s. The percent error tended to be higher for the GPS60 and lower for the DG100 on both sides of the x-axis. The percent error was significantly lower for the DG100 as compared to the GPS60 (non-parametric test, *P*<0.001).

### Experiment 2

The aim was to determine the accuracy of the DG100 when estimating the distance and speed of outdoor walking from other PWPs. The accuracy of the DG100 was compared to the accuracy of the GPS60. A sufficient number of satellite signals were always available for the DG100. For the GPS60, only one episode of undetectable satellite signal that lasted 2.72 min was noted during one PWP, leading to the exclusion of two bouts of walking from the statistical analysis. Mean ± standard deviation of walking speed for the DG100 was 3.2±0.9 km/h (range 1.4 to 4.8 km/h) for “slow” pace series, and was 5.5±0.6 km/h (range 4.1 to 6.8 km/h) for “usual” pace series. Table 1 presents the TEE and CV of the estimated walking distances and speeds for each GPS device. As shown, when considering all pooled distances the values for the DG100 were better (lower CV) when compared to those of the GPS60 (*p*<0.001).

**Table 1:** Accuracy of the estimation of processed walking distances and speeds, according to the covered distance, for both the DG100 and the GPS60; the typical error of the estimate (TEE) and the coefficient of variation (CV) with 95% Confidence Interval (95% CI) are presented.

	Accuracy of the estimation of processed walking distances^1^				Accuracy of the estimation of processed walking speeds^2^			
Distances (m)	TEE [95% CI]		CV [95% CI]		TEE [95% CI]		CV [95% CI]	
	DG100	GPS60	DG100	GPS60	DG100	GPS60	DG100	GPS60
50	2.0 [1.6–2.6]	4.7 [3.8–6.0]	4.2 [3.4–5.4]*	9.7 [7.9–12.7]	0.1 [0.1–0.2]	0.4 [0.4–0.6]	3.4 [2.8–4.4]*	8.9 [7.3–11.6]
100	4.7 [3.8–6.0]	5.4 [4.4–7.0]	5.0 [4.1–6.4]	5.4 [4.4–7.0]	0.2 [0.2–0.2]	0.2 [0.2–0.3]	4.6 [3.8–6.0]	4.5 [3.6–5.8]
150	5.2 [4.2–6.6]	9.9 [8.1–12.7]	3.5 [2.9–4.6]*	6.2 [5.1–8.1]	0.1 [0.1–0.1]	0.2 [0.1–0.2]	3.4 [2.7–4.3]*	5.6 [4.6–7.2]
200	4.2 [3.4–5.3]	8.3 [6.8–10.7]	2.1 [1.7–2.7]*	4.0 [3.3–5.2]	0.1 [0.0–0.1]	0.1 [0.1–0.1]	1.7 [1.4–2.1]*	3.6 [2.9–4.6]
250	5.9 [4.8–7.5]	7.3 [6.0–9.4]	2.4 [2.0–3.1]	2.9 [2.3–3.7]	0.1 [0.1–0.1]	0.1 [0.1–0.2]	2.2 [1.8–2.8]	2.6 [2.1–3.4]
300	6.6 [5.4–8.5]	8.8 [7.2–11.4]	2.2 [1.8–2.9]	2.9 [2.4–3.7]	0.1 [0.1–0.1]	0.1 [0.1–0.2]	2.2 [1.8–2.8]	2.6 [2.1–3.4]
350	7.1 [5.8–9.1]	9.7 [8–12.6]	2.0 [1.7–2.6]*	2.7 [2.2–3.5]	0.1 [0.1–0.1]	0.1 [0.1–0.2]	2.4 [1.9–3.0]*	3.3 [2.7–4.3]
400	6.9 [5.7–8.9]	12.9 [10.6–16.5]	1.8 [1.4–2.3]*	3.2 [2.6–4.1]	0.1 [0.1–0.1]	0.1 [0.1–0.2]	1.7 [1.4–2.2]*	3.1 [2.6–4.0]
All pooled distances (mean distance = 225 m)	5.6 [5.2–6.1]	8.9 [8.3–9.7]	3.1 [2.9–3.3]*	5.1 [4.7–5.5]	0.1 [0.1–0.1]	0.2 [0.2–0.2]	2.8 [2.6–3.1]*	4.7 [4.3–5.1]

1 For the accuracy of the estimation of processed walking distances, TEE is expressed in meters and CV is expressed in percentage.

2 For the accuracy of the estimation of processed walking speeds, TEE is expressed in km.h^−1^ and CV is expressed in percentage.

For CV values comparisons: * is significantly different from GPS60 (*P*<0.05).

## Discussion

This study provides original results about the accuracy of a handy GPS data logger/receiver, the DG100, in order to study outdoor human walking for health and clinical research studies. There were two major findings of the present study. First, the DG100 is accurate for the detection of bouts of walking and resting. Second, it estimates with accuracy the speed and distance of walking. It should be noted that in all cases, the DG100 performed better than the GPS60. Although it was not a primary goal of the study, this is of particular interest because the data processing that was used here was previously developed and validated for the GPS60 device. This study confirms the robustness of the previously validated processing method and its external validity.

### Accuracy of the DG100 GPS to study human outdoor walking

#### The detection of bouts of walking and resting

Surprisingly, very few studies have addressed this issue or the detection of other physical activities using the GPS. However, when quantifying a physical activity, or more specifically, when studying outdoor walking as in the present study, accurately detecting periods of walking is a major issue and a necessary first step, particularly for medical applications [11], [12], [15]. Troped *et al.* [23] showed that the addition of GPS to accelerometer monitoring slightly improved the classification of physical activity, particularly for walking. A direct comparison of our results with the study of Troped *et al.* remains difficult because i) the GPS data analysis was performed from the GPS dataset composed of minute-by-minute observations (every 2 s here) and ii) the authors only focused on the detection of bouts of walking.

The results of the present study can be compared directly to our previous study that focused on the accuracy of the GPS60 for the detection of bouts of walking and resting [15]. We reported the accuracy at the bout level to be 97.1% (95% CI, 93.5–98.8) using the same study design and the same GPS data processing methodology [15]. This is considerably higher than the accuracy that was reported in the present study. This is not surprising because the PWPs that were performed in the present study voluntarily included a high proportion of short bouts as compared to our previous study [15], and errors in the detection of bouts arise primarily from the incorrect detection of shorts bouts (Figure 2).

#### The estimation of walking distances and speeds

It should be pointed out that the present study aimed to assess the accuracy of the GPS for the estimation of walking distances and speeds and not to assess the positional accuracy (*i.e.*, statistical accuracy), which is of little interest in view of our final clinical application. The results of the accuracy of estimating walking distances and speeds that are reported in the present study are within the range of the results that are reported in validation studies that focused specifically on walking and that have been carried out since the selective availability was turned off in May 2000 [15], [16], [24], [25], [26]. Selective availability refers to the automatic and deliberate degradation of the satellites' signals by the U.S. Department of Defense before May 2000. Using a nondifferential GPS device during 40 bouts of walking that were 100 m long, Townshend *et al.* [16] reported an error in the estimation of walking distances less than 0.50 m. As compared to both our present and our previous study [15], in which we also performed bouts of walking that were 100 m long, the accuracy reported by Townshend *et al.* was particularly high. However, this high accuracy in estimating the distances of walking did not translate into a particularly high accuracy for the estimation of speeds (walking and running) as compared to the other available studies [15], [25].

Others studies that were designed for sports applications have used sport-specific GPS devices to assess different movement patterns, including walking activity [27], [28], [29]. It should be noted that most of these studies used relatively expensive devices that analyzed a combination of GPS-signal and accelerometry. These devices used algorithms that retrieve data from the built-in high-frequency accelerometer to correct 1 Hz or 5 Hz GPS values, which is expected to improve the accuracy of the GPS devices. Consequently, it is difficult to comment on the “real” accuracy of the GPS devices for the estimation of speed and distance. Using the WI SPI elite GPS (GPSports, Canberra, ACT, Australia), Gray *et al.* [29] reported a mean total distance error of 5.8 m when walking over a straight 200-m path, which is consistent with the results that were reported in the present study with the DG100 (Table 1). Interestingly, over very short distances of 10, 20 and 40 m, Jennings *et al.* [28] studied the validity of using two MinimaxX GPS devices simultaneously, with sampling rates of 1 Hz and 5 Hz respectively. With the 1 Hz sampling rate, the authors reported a standard error of the estimate of 23.8, 17.4 and 9.6% for 10, 20 and 40 m, respectively. With the 5 Hz device, the authors reported CVs of 21.3, 16.6 and 9.8% for 10, 20 and 40 m, respectively. These results indicate that there is probably little benefit to using a GPS device with a sampling rate higher than 1 Hz if only walking locomotion is studied. For instance, in the present study, using the DG100 sampling at 0.5 Hz, we found a CV of 4.2% for a 50 m walking distance estimation. Finally, over a walking distance of 8800 m, Petersen *et al.* [27] reported a standard error of the estimate ranging between 0.6% and 3.8%, for the five GPS devices that were tested.

### DG100 *vs.* GPS60: technical considerations for the user

Experiments 1 and 2 clearly indicated that the DG100 was more accurate than the GPS60. Although we did not specifically study the reasons for such differences, the comparison shows that the GPS model used has important consequences. One potential explanation is the type of chipset that was present in the GPS devices. The GPS60 is built with a SiRF II chipset, whereas the DG100 is built with a SiRF III chipset. One of the expected advantages of the SiRF III chipset is a higher sensitivity for improved locking of satellite signals in low signal areas and this could explain the higher accuracy that was found for the DG100 in this study. Thus, when possible, users should prefer GPS devices that are built with the more recent chipsets, such as the SiRF III chipset. A possible consequence of this technological difference is the lower variation in walking speeds that was observed with the DG100 (Figure 1). It is likely that wider variation in walking speeds led to a high proportion of false resting bouts, thus decreasing the accuracy of detection of the GPS60, particularly when the walking speed was slow. When we calculated the coefficient of variation of the processed walking speeds among each bout of walking that was accurately detected, we found a wider variation of walking speeds for the GPS60 as compared to the DG100 (results not shown).

However, it is important to realize that the accuracy of the GPS does not only depend on the type of chipset. The precision of the algorithm used by the software to convert and correct raw data probably also accounts for an important part in the accuracy of a GPS device. These algorithms are, unfortunately, considered to be proprietary.

The fact that the accuracy of the GPS60 was considerably lower than the DG100 does not imply that it is not accurate for the study of outdoor walking. The accuracy of the GPS60 is lowest on shorts bouts (*i.e.* bouts ≤30 s and particularly bouts ≤15 s), as previously reported [15]. Depending on the final application, such shorts bouts of walking and/or resting, may, or may not be of clinical interest. For instance, most patients with vascular intermittent claudication have stops induced by lower-limb pain that last approximately 2 min [12]. However, in patients with impaired ambulation from hemiparetic stroke, the ability to perform short bouts of walking >5 steps can be an outcome measure that is assessed [30]. Thus, in such clinical context, the DG100 should be preferred.

Other important considerations for users when using a GPS device are the choice of the sampling rate when configuring the GPS and the use of the WAAS/EGNOS function. In the present study, we used a sampling rate of 0.5 Hz, as in our previous studies [11], [12], [15]. It is important to note that the processing methodology of the GPS speed signals we used for the detection of bouts of walking and non-walking was initially developed with a sampling rate of 0.5 Hz [15]. If required, this methodology could be used with a 1 Hz sampling rate. For slow displacements such as walking, a 0.5 Hz sampling rate is sufficient. However, if one would assess bursts displacements over bouts of short duration, a sampling rate of 1 Hz and higher should be preferred.

The WAAS/EGNOS function improves the determination of position by collecting error-correction data from multiple reference stations *via* terrestrial communications [31], [32]. The error-correction data are transmitted to an additional “geostationary earth orbit satellite”, after which they are re-transmitted to the GPS receiver. The WAAS system works in North America, whereas the EGNOS system works in Western Europe. According to the Federal Aviation Administration (http://www.faa.gov), the accuracy in positional estimation using the WAAS system is approximately seven meters. The official EGNOS Portal (http://egnos-portal.gsa.europa.eu) reports that an EGNOS-enabled receiver can provide location accuracy to within three meters. This is the accuracy that is typically reported by GPS manufacturers. However, there is a lack of studies examining the accuracy of a WAAS-enabled GPS unit for determining the position, speed and distance of human locomotion. To the authors' knowledge, only Witte and Wilson have addressed this issue, although they focused only on cycling [32], [33]. The authors reported the distribution of error values and showed that 81% of the values recorded by the WAAS-enabled unit were within 0.4 m.s^−1^ *vs*. 64% for the non-WAAS unit [32], .

Lastly, two important points need to be considered for health and clinical research in large cohort studies: the cost and the practicality of the device. Because of its very low cost (below $100) and its ease of use (only one start/stop button and signals acquisition is indicated by a flashing LED), the DG100 is a good candidate for such studies.

### Conclusion

The results of this study indicate that the DG100 produces acceptable accuracy both in detecting bouts of walking and resting and in estimating distances and speeds of walking during the detected periods of walking. Our results show that the accuracy of the DG100 was better than the accuracy of the GPS60, a somewhat older GPS device dating back to ∼1996. This additional result suggests that the rapid innovation of GPS technology is likely to improve the performance of new GPS devices as compared to older GPS devices, and at a lower cost. Further studies are needed to address the inter- and intra-DG100 variability.
